# Identification of Anti-Melanogenesis Constituents from *Morus alba* L. Leaves

**DOI:** 10.3390/molecules23102559

**Published:** 2018-10-08

**Authors:** Hong Xu Li, Jung Up Park, Xiang Dong Su, Kyung Tae Kim, Jong Seong Kang, Young Ran Kim, Young Ho Kim, Seo Young Yang

**Affiliations:** 1 College of Pharmacy, Chungnam National University, Daejeon 34134, Korea; charon0077@gmail.com (H.X.L.); suxiangdongnicky@163.com (X.D.S.); kangjss@cnu.ac.kr (J.S.K.); 2 College of Pharmacy and Research Institute of Drug Development, Chonnam National University, Gwangju 61186, Korea; gk2560@naver.com; 3 Division of Applied Bioengineering, College of Engineering, Dong-Eui University, Busan 47340, Korea; kimkkt@deu.ac.kr

**Keywords:** *Morus alba* leaf, anti-melanogenesis, MITF, tyrosinase, TRP-1, TRP-2

## Abstract

The individual parts of *Morus alba* L. including root bark, branches, leaves, and fruits are used as a cosmetic ingredient in many Asian countries. This study identified several anti-melanogenesis constituents in a 70% ethanol extract of *M. alba* leaves. The ethyl acetate fraction of the initial ethanol extract decreased the activity of tyrosinase, a key enzyme in the synthetic pathway of melanin. Twelve compounds were isolated from this fraction and their structures were identified based on spectroscopic spectra. Then, the authors investigated the anti-melanogenesis effects of the isolated compounds in B16-F10 mouse melanoma cells. Compounds **3** and **8** significantly inhibited not only melanin production but also intracellular tyrosinase activity in alpha-melanocyte-stimulating-hormone (α-MSH)-induced B16-F10 cells in a dose-dependent manner. These same compounds also inhibited melanogenesis-related protein expression such as microphthalmia-associated transcription factor (MITF), tyrosinase, and tyrosinase-related protein-1 (TRP-1). Compound **3** modulated the cAMP-responsive element-binding protein (CREB) and p38 signaling pathways in α-MSH-activated B16-F10 melanoma cells, which resulted in the anti-melanogenesis effects. These results suggest that compound **3**, isolated from *M. alba* leaves, could be used to inhibit melanin production via the regulation of melanogenesis-related protein expression.

## 1. Introduction

Melanin plays an important role in determining the color of human skin and hair. It also protects cells by absorbing ultraviolet radiation and exhibits remarkable antioxidant effects [1]. However, the abnormal overproduction of melanin in skin can cause dots, freckles, bruising keratosis, and hyperpigmentation [2,3]. Melanin is produced by melanocytes in the basal layer of the skin epidermis. Melanogenesis occurs through the activation of several melanogenesis-related proteins, including microphthalmia-associated transcription factor (MITF), tyrosinase, tyrosinase-related protein-1 (TRP-1), and tyrosinase-related protein-2 (TRP-2) [4]. MITF is an essential transcription factor for melanogenesis in differentiated melanocytes and is involved in the expression of tyrosinase. Tyrosinase, TRP-1, and TRP-2 are key enzymes in the synthetic pathway of melanin. Tyrosinase converts l-tyrosine into 3, 4-dihydroxyphenylalanine (DOPA). DOPA quinone is generated by the oxidation of DOPA, which ultimately produces eumelanin and pheomelanin via catalysis with pigmentary enzymes such as glutathione, TRP-1, and TRP-2 [5]. In melanocyte cells, the cyclic adenosine monophosphate (cAMP)/protein kinase A (PKA) cascades are known to play crucial roles in melanogenesis. Alpha-melanocyte-stimulating-hormone (α-MSH) causes an increase in intracellular cAMP concentration and the activation of the PKA cascade. Then, PKA phosphorylates the cAMP-responsive element-binding protein (CREB), which in turn activates the promoter of MITF [6,7].

*Morus alba* L., also known as mulberry, is widely cultivated in several Asian countries, including China, India, Japan, and Korea. In traditional Chinese medicine, *M. alba* leaves have been used to treat fever and cold for thousands of years. It has been reported to exhibit various biological activities, including anti-inflammatory, neuroprotection, antioxidant, anti-Alzheimer’s, and anti-diabetic effects [8,9,10,11,12]. Recently, extracts from *M. alba* leaves have been shown to inhibit melanogenesis in mushroom tyrosinase activity assays [13,14]. In this study, the authors found that the ethyl acetate fraction from a 70% ethanol extract of *M. alba* leaves decreased the activity of tyrosinase, a key enzyme in the synthetic pathway of melanin. In addition, they isolated 12 compounds from this extract and evaluated their anti-melanogenesis effects by monitoring the melanin content, intracellular tyrosinase activity, and MITF expression in α-MSH-stimulated B16-F10 cells.

## 2. Results and Discussion

**Figure 1:**
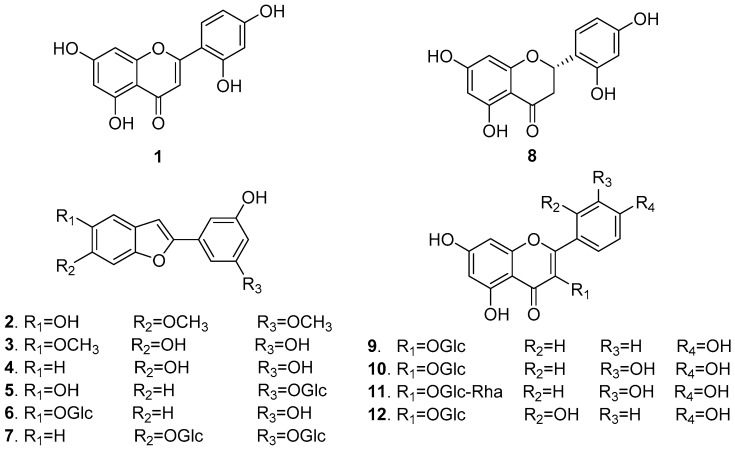
Structures of compounds **1**–**12** from *M. alba* leaves.

**Figure 2:**
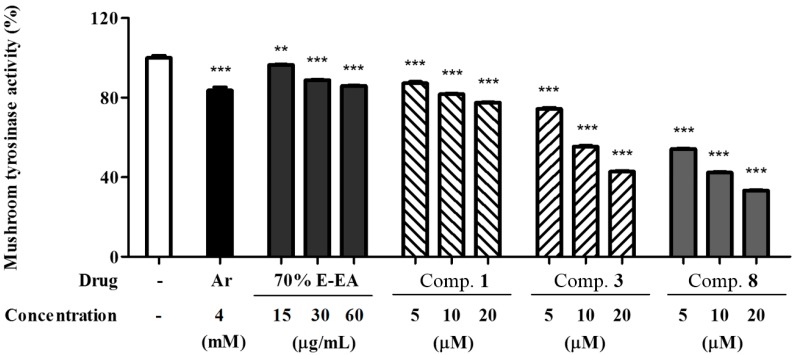
Inhibitory effects of *M. alba* leaves on mushroom tyrosinase activity; a mushroom tyrosinase activity was assayed by measuring 3,4-dihydroxyphenylalanine (DOPA) oxidase activity. The test samples were diluted in sodium phosphate buffer (67 mM, pH 6.8) and added to the samples in 96-well plates. Then, l-dopa (2 mM) and mushroom tyrosinase (200 U) were added to each well for 10 min. The absorbance was measured at 490 nm by using an ELISA microplate reader. Results are expressed as percentages of ** *p* < 0.01 and *** *p* < 0.001 compared with the control group. 70% E-EA indicates ethyl acetate fraction from 70% ethanol extract of *M. alba* leaves.

In mammals, melanin plays a crucial role in protecting skin against photocarcinogenesis [15]. However, various stimuli such as the abnormal release of α-MSH, oxidative stress, ultraviolet radiation, and skin rubs can affect the melanin content of cells (i.e., pigmentation) [16]. To identify anti-melanogenesis constituents in *M. alba*, the authors isolated 12 components from a 70% EtOH extract of *M. alba* leaves. These included six moracin derivatives, four flavonol glycosides, one flavone, and one flavanone. These compounds were identified as norartocarpetin (**1**) [17], moracin B (**2**) [18], moracin J (**3**) [19], moracin M (**4**) [17], moracin M 3′-*O-*β-glucopyranoside (**5**) [20], moracin M 6-*O*-β-d-glucopyranoside (**6**) [21], mulberroside F (**7**) [22], steppogenin (**8**) [23], astragalin (**9**) [24], isoquercitrin (**10**) [25], rutin (**11**) [26], and morin 3-*O-*β-d-glucopyranoside (**12**) [27] by comparing their NMR spectroscopic data with previously published reports (see Figure 1, Supplementary Materials). Mushroom tyrosinase is widely used to screen for potential inhibitors of melanogenesis. Among the isolates, compounds **1**, **3**, and **8** significantly inhibited mushroom tyrosinase in a dose-dependent manner (see Figure 2).

**Figure 3:**
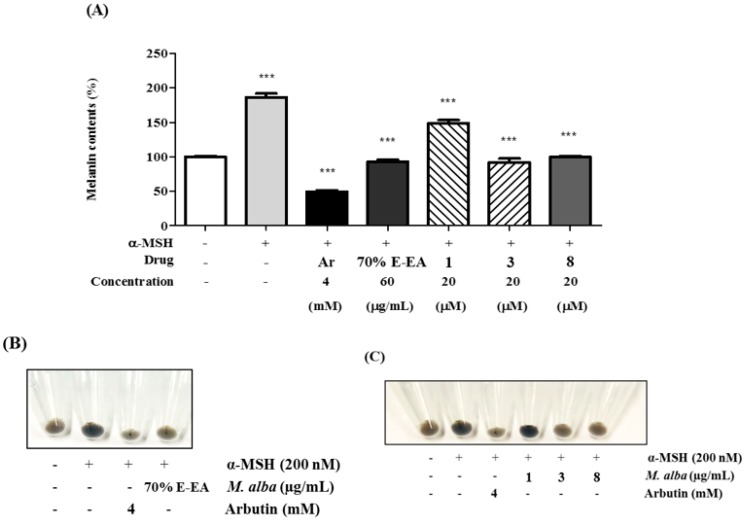
Effects on melanogenesis in murine B16-F10 cells; B16-F10 cells were pretreated with test samples for 2 h and then incubated with alpha-melanocyte-stimulating-hormone (α-MSH) (200 nM) for 72 h. The cell pellets were dissolved in 1N NaOH with 10% DMSO. The cell lysates were transferred to 96-well plates and the absorbances at 405 nm were detected using an ELISA microplate reader. (**A**) 70% E-EA, **1**, **3**, and **8** decreased the α-MSH-induced production of melanin. *** *p* < 0.001 compared with the α-MSH-treated group; (**B**,**C**) indicate the colors of the pellets from α-MSH-induced B16-F10 melanoma cells.

Consequently, compounds **1**, **3**, and **8** were evaluated for inhibitory effects on melanin production in α-MSH-stimulated B16-F10 melanoma cells. Compounds **3** and **8** markedly decreased the production of melanin (see Figure 3). The anti-melanogenesis effects of compounds **3** and **8** were greater than that of compound **1** (see Figure 3A). In comparing the color of the cell pellets, it was clear that compounds **3** and **8** inhibited melanin formation (see Figure 3B,C). 

**Figure 4:**
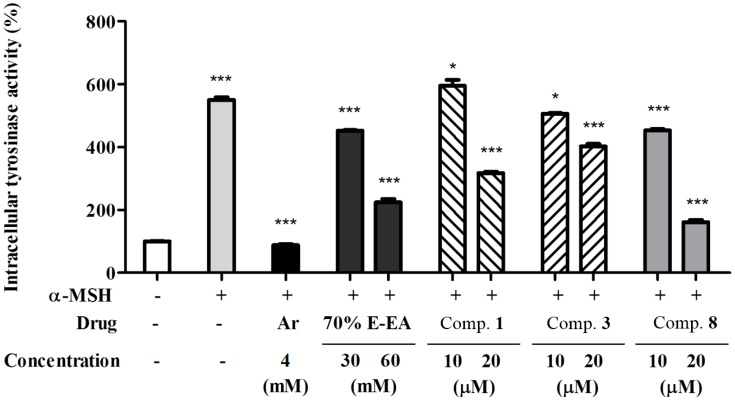
Effects on intracellular tyrosinase activity in murine B16-F10 cells; B16-F10 cells were pretreated with test samples for 2 h and then incubated with α-MSH (200 nM) for 72 h. The cell lysates (50 μg) were dissolved in 100 mM phosphate buffer (pH 6.8) and treated with L-dopa (2 mg/mL) in a 96-well plate at 37 °C for 2 h. The production amount of dopachrome was measured by using an ELISA microplate reader at 490 nm. * *p* < 0.05 and *** *p* < 0.001 compared with the α-MSH-treated group.

In addition, the ethyl acetate fraction of the initial ethanol extract (70% E-EA) and the three single compounds isolated from it were evaluated for their inhibitory effects against intracellular tyrosinase activity in α-MSH-induced B16-F10 melanoma cells. Compounds **3** and **8** significantly inhibited intracellular tyrosinase activity in a dose-dependent manner in α-MSH-stimulated B16-F10 cells (see Figure 4). 

**Figure 5:**
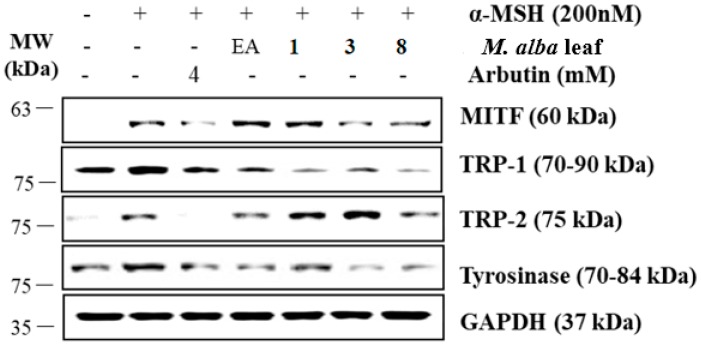

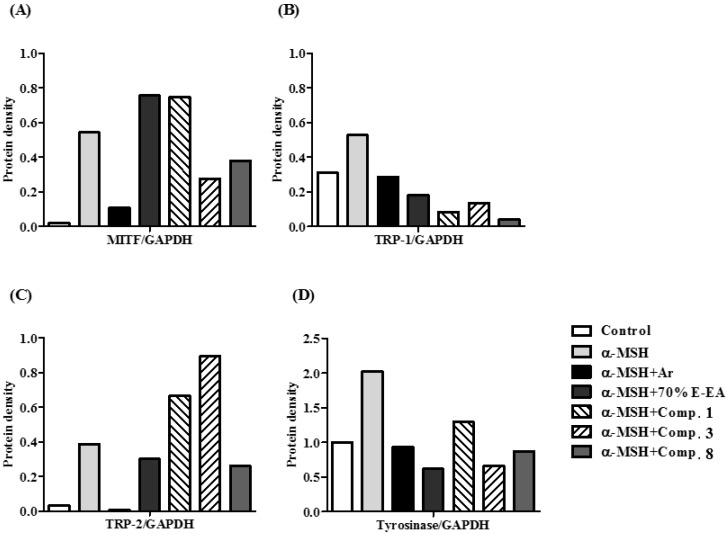
Effects on melanogenesis-related protein expression in α-MSH-induced murine B16-F10 cells; B16-F10 cells were pretreated with test samples for 2 h and α-MSH was added to the cells for 24 h. Glyceraldehyde 3-phosphate dehydrogenase (GAPDH) was used as the control protein. The protein levels of microphthalmia-associated transcription factor (MITF), tyrosinase, tyrosinase-related protein-1 (TRP-1), and tyrosinase-related protein-2 (TRP-2) were detected by Western blot analysis. Compounds **3** and **8** significantly reduced the expression of MITF, tyrosinase, TRP-1, and TRP-2 in α-MSH-induced B16-F10 cells. Arbutin, a positive control drug, also blocked the melanogenesis. (**A**) MITF, (**B**) TRP-1, (**C**) TRP-2, and (**D**) tyrosinase were analyzed and quantified using Azure Spot software. MITF, TRP-1, TRP-2 and tyrosinase protein levels were normalized by GAPDH.

Finally, to determine the mechanism of inhibition, the expressions of MITF, tyrosinase, TRP-1, and TRP-2 were determined by Western blot analyses using the cell lysates of α-MSH-induced B16-F10 cells. MITF, a leucine zipper transcription factor, binds to the M-box and activates the expression of multiple genes for enzymes important in the conversion of tyrosine into melanin, resulting in increased levels of these melanin-producing proteins [28]. Two tyrosinase derivatives, TRP-1 and TRP-2, are melanocyte-specific enzymes involved in melanogenesis. Compounds **3** and **8** significantly inhibited the expression of both transcription factor MITF and downstream enzymes such as tyrosinase, TRP-1, and TRP-2 (see Figure 5). Compound **3** exhibited a much higher degree of melanogenesis inhibition than the other compounds.

**Figure 6:**
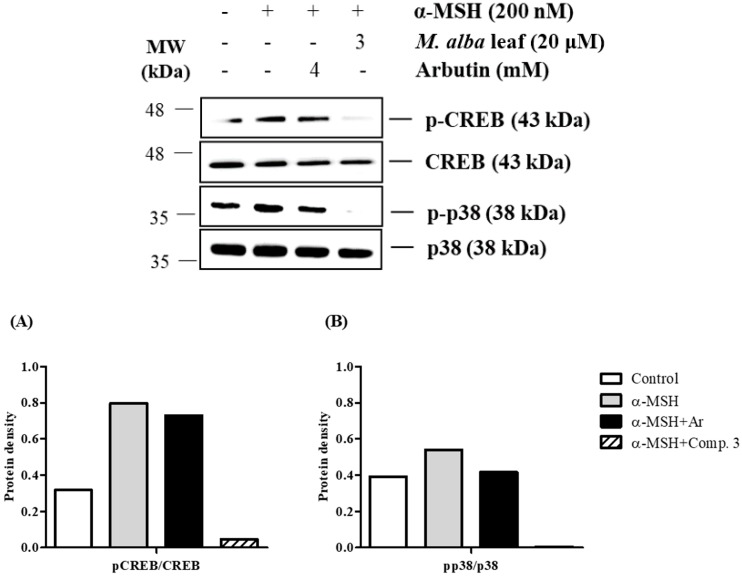
Effects of *M. alba* leaves on the phosphorylation of the cAMP-responsive element-binding protein (CREB) and p38 in α-MSH-induced B16-F10 cell; B16-F10 cells were pretreated with test samples for 2 h and α-MSH was added to the cells for 24 h. Total protein was used as the control protein. The protein levels of pCREB, CREB, pp38, p38 were detected by Western blot analysis. Compound **3** significantly reduced the expression of pCREB and pp38 in α-MSH-induced B16-F10 cells. Arbutin, a positive control drug, also blocked the melanogenesis. The protein level of (**A**) pCREB and (**B**) pp38 were quantitated with Azure Spot software. pCREB and pp38 protein levels were normalized by the total protein of CREB or p38.

The results from this study showed that compound **3** had the most anti-melanogenesis effect in α-MSH-induced B16-F10 cells. To study the action mechanism of compound **3**, the authors investigated the phosphorylation of the proteins involved, CREB and mitogen-activated protein kinases (MAPKs), by using Western blot analysis. B16-F10 cells were treated with the compound **3** or arbutin (4 mM) and activated with α-MSH (200 nM) for 24 h. As shown in Figure 6, the phosphorylation of CREB and p38 proteins was significantly decreased by the treatment with compound **3** in α-MSH-induced B16-F10 cells. In contrast, the phosphorylation of extracellular signal-regulated kinase (ERK) 1/2 and c-Jun N terminal kinase (JNK) proteins was not increased by the treatment with compound **3** in B16-F10 cells (data not shown). These results indicated that the suppressive mechanism of melanogenesis by compound **3** was related to the inhibition of CREB and p38 signaling pathways. 

In this study, six 2-arylbenzofuran derivatives (**2**–**7**) were isolated from *M. alba* leaves. 2-Arylbenzofuran derivatives usually lead to substantially weakened tyrosinase inhibition due to the formation of five-membered rings in the structure [29,30]. Furthermore, the resorcinol moiety of 2-arylbenzofuran derivatives may result in decreased tyrosinase inhibition. These data may explain the lack of tyrosinase inhibition exhibited by compounds **2** and **4**–**7** [19,23]. Remarkably, moracin J (**3**) was a potent inhibitor of tyrosinase and significantly reduced melanin production in α-MSH-stimulated B16-F10 melanoma cells. This implies a structure–activity relationship between the 2-arylbenzofuran derivatives found in *M. alba* leaves and tyrosinase inhibition. Compounds **1** and **8** exhibited significant anti-melanogenesis effects, whereas compounds **9**–**12**, which all possess sugar units at the C-3 position of the C-ring, showed no observable inhibition of tyrosinase. Previous reports have shown that substitutions, including glucose groups at the C-3 position of the C-ring, effectively attenuate the inhibition of tyrosinase activity [29]. Moreover, compounds **1** and **8** were structurally similar with hydroxyl groups at the C-2′ and C-4′ positions of the B-ring and at C-5 and C-7 of the A-ring. Thus, tyrosinase inhibition by compounds **1** and **8** may be attributed to this unique structural feature [23,29]. However, further research is required to fully elucidate the structure–activity relationships between compounds **1** and **8** and their effects on tyrosinase activity.

## 3. Materials and Methods

### 3.1. General Information

The NMR spectra were recorded using a BRUKER AVANCE III 600 (Bruker Biospin GmbH, Karlsruhe, Germany), with tetramethylsilane (TMS) as an internal standard. The ESI-MS spectra were obtained by using an Agilent 1200 LC-MSD Trap spectrometer (Agilent, Santa Clara, CA, USA). Preparative HPLC was performed using a GILSON 321 pump, 151 UV/VIS detector (Gilson, Villiers-le-Bel, France), and RS Tech HECTOR-M C18 column (5 micron, 250 × 21.2 mm, RS Tech Crop, Chungju, South Korea). Column chromatography was performed using a silica gel (Kieselgel 60, 70–230, and 230–400 mesh, Merck, Darmstadt, Germany), YMC C-18 resins, and thin-layer chromatography (TLC) was performed using pre-coated silica-gel 60 F254 and RP-18 plates (both 0.25 mm, Merck, Germany), and the spots were detected under UV light and using 10% H_2_SO_4_.

### 3.2. Plant Material

Dried leaves of *M. alba* L. were purchased from the herbal company Naemome Dah, Ulsan, Korea, in September 2015, and identified by Prof. Young Ho Kim, College of Pharmacy, Chungnam National University. A voucher specimen (CNU 16004-1) was deposited at the Herbarium of College of Pharmacy, Chungnam National University, Daejeon, Korea.

### 3.3. Extraction and Isolation

The dried leaves of *M. alba* (2.1 kg) were extracted three times with 70% EtOH under reflux. The 70% EtOH extract (243.0 g) was suspended in deionized water and partitioned with *n*-hexane, EtOAc, and *n*-BuOH to yield *n*-hexane fraction (1A, 86.0 g), EtOAc fraction (1B, 10.1g), *n*-BuOH fraction (1C, 40.0 g), and aqueous fraction (1D, 94.0 g), respectively. The EtOAc fraction was subjected to a silica gel column chromatography with a gradient of *n*-hexane-EtOAc (9:1, 8:2, 7:3, 5:5, 4:6, 3:7, 2:8, and 100% MeOH) to give eight fractions (1B-1–1B-8). The fraction 1B-1 was chromatographed with a gradient of MeOH: water (1:4, 1:3, 1:2, 1:1, and MeOH) by MPLC using C_18_ column to give four fractions (1B-1-1–1B-1-4). Subfraction 1B-1-4 was separated by a sephadex LH-20 column and eluted by MeOH and its subfraction was isolated by prep-HPLC to give compound **2** (6.1 mg). The fraction 1B-4 was isolated with a gradient of MeOH: water (1:4, 1:3, 1:2, 1:1, and MeOH) by MPLC using C_18_ column to give 3 fractions (1B-4-1–1B-4-3). Subfraction 1B-4-2 was separated by a sephadex LH-20 column and eluted by MeOH and its subfraction was isolated by prep-HPLC to give compound **1** (5.9 mg). The fraction 1B-5 was isolated with a gradient of MeOH: water (1:3, 1:2, 1:1, and MeOH) by MPLC using C_18_ column to give four fractions (1B-5-1–1B-5-4). Subfraction 1B-5-2 was separated by a sephadex LH-20 column and eluted by MeOH and its subfraction was isolated by prep-HPLC to give compound **8** (9.3 mg) and compound **3** (6.5 mg). Subfraction 1B-5-4 was separated by a sephadex LH-20 column and eluted by MeOH and its subfraction was isolated by prep-HPLC to give compound **9** (30.0 mg) and compound **10** (9.7 mg). The fraction 1B-8 was isolated with a gradient of MeOH: water (1:2, 1:1, and MeOH) by MPLC using C_18_ column to give nine fractions (1B-8-1–1B-8-9). Subfraction 1B-8-7 was separated by a sephadex LH-20 column and eluted by MeOH and its subfraction was isolated by prep-HPLC to give compound **4** (20.1 mg). Subfraction 1B-8-8 was separated by a sephadex LH-20 column and eluted by MeOH and its subfraction was isolated by prep-HPLC to give compound **6** (9.7 mg). Subfraction 1B-8-9 was separated by a sephadex LH-20 column and eluted by MeOH and its subfraction was isolated by prep-HPLC to give compound **5** (6.1mg). The *n*-BuOH fraction was subjected to a silica gel column chromatography with a gradient of CHCl_3_: MeOH: water (10:1:0, 9:1:0, 8:1:0, 6:1:0.1, 5:1:0.1, 4:1:0.1, 3:1:0.1, 2:1:0.1, and 100% MeOH) to give nine fractions (Fr. 1C-1–1C-9). The fraction 1C-4 was isolated with a gradient of MeOH: water (1:3, 1:2, 1:1, and MeOH) by MPLC using C_18_ column to give five fractions (1C-4-1–1C-4-5). Subfraction 1C-4-4 was separated by a sephadex LH-20 column and eluted by MeOH and its subfraction was isolated by prep-HPLC to give compound **12** (18.5 mg). The water fraction was subjected to an HP-20 column, and eluted with water, 25% MeOH, 50% MeOH, 75% MeOH, and 100% MeOH, yielding five fractions (1D-1–1D-5). Fraction 1D-2 and 1D-3 were combined (1D-2-1), and isolated with a gradient of MeOH: water (1:4, 1:3, 1:2, 1:1, and MeOH) by MPLC using C_18_ column to give five fractions (1D-2-1-1–1D-2-1-5). Subfraction 1D-2-1-2 was separated by a sephadex LH-20 column and eluted by MeOH and its subfraction was isolated by prep-HPLC to give compound **7** (17.8 mg). Sub-fraction 1D-2-1-3 was separated by a sephadex LH-20 column and eluted by MeOH and its subfraction was isolated by prep-HPLC to give compound **11** (10.0 mg).

### 3.4. Cell Cultures

B16-F10 mouse skin melanoma cells provided from the Korea Cell Line Bank (Seoul, Korea) were cultured in Dulbecco′s Modified Eagle’s Medium (DMEM) (Welgene, Daegu, Korea) supplemented with 1% penicillin/streptomycin (Thermo Fisher Scientific, Waltham, MA, USA) and 10% heat-inactivated fetal bovine serum (FBS) (Thermo Fisher Scientific, Waltham, MA, USA) in a humidified 37 °C incubator with 5% CO_2_. 

### 3.5. Measurement of Mushroom Tyrosinase Activity

A mushroom tyrosinase activity assay was conducted by measuring DOPA oxidase activity as previously described [31]. Briefly, the test samples were diluted in a sodium phosphate buffer (67 mM, pH 6.8) and dispensed in each well. The reagents and samples used were arbutin (4 mM) (Sigma-Aldrich Chemical Co, St. Louis, MO, USA), the ethyl acetate fraction from 70% ethanol extract of *M. alba* leaves (70% E-EA fraction) (60, 30, 15 μg/mL), and three compounds (20, 10, 5 μM). Then, the L-dopa (2 mM) (Sigma-Aldrich Chemical Co, St. Louis, MO, USA) was combined with the samples, and mushroom tyrosinase (200 U) (Sigma-Aldrich Chemical Co, St. Louis, MO, USA) was then added to each well for 10 min. The absorbance was measured at 490 nm by using an ELISA microplate reader (ELx808, BioTek Instruments, Inc., Winooski, VT, USA).

### 3.6. Measurement of Melanin Contents in B16-F10 Cells

The melanin contents were measured using a previously described method with slight modifications [32,33]. B16-F10 cells (1 × 10^5^/well) were seeded into a 6-well plate (SPL Life Sciences Co., Pocheon, Korea) and then incubated for 24 h. The culture medium was removed and replaced with fresh DMEM medium with 10% FBS. The cells were pretreated with 70% E-EA fractions (60 µg/mL) and three compounds (20 µM), or arbutin (4 mM) for 2 h, and then α-MSH (200 nM) (Sigma-Aldrich Chemical Co, St. Louis, MO, USA) was added to the cells for 72 h. The cells were washed with cold phosphate-buffered saline (PBS) and dissolved in 1N NaOH with 10% DMSO for 2 h at 60 °C. The cell lysates were transferred to a 96-well plate and the optical absorbance was measured at 405 nm using an ELISA microplate reader.

### 3.7. Assay of Intracellular Tyrosinase Activity

The intracellular tyrosinase activity was determined as described in an article with some modifications [32,34]. B16-F10 cells (1 × 10^5^/well) were treated with 70% E-EA (60, 30 µg/mL), three compounds (20, 10 µM), or arbutin (4 mM) for 2 h, followed by the addition of α-MSH (200 nM) for 72 h. The cells were lysed in a lysis buffer (Promega, Madison, WI, USA) with protease inhibitor cocktail (PIC) for 30 min at 4 °C and then centrifuged at 13,000 RPM for 10 min at 4 °C. The lysates (50 μg) were dissolved in 100 mM phosphate buffer (pH 6.8) and treated with L-dopa (2 mg/ml) in a 96-well plate at 37 °C for 2 h. The production amount of dopachrome was measured using an ELISA microplate reader (ELx808) at an absorbance of 490 nm.

### 3.8. Western Blot Analysis

B16-F10 cells were cultured at a density of 1.5 × 10^5^ cells/well in 6-well plates and pretreated with 70% E-EA (60 µg/mL), three compounds (20 µM), or arbutin (4 mM) for 2 h, followed by treatment with α-MSH (200 nM) for 24 h. The cells were washed twice with cold PBS and lysed in a lysis buffer with PIC for 30 min on ice. The cell lysates were centrifuged at 12,000 RPM for 10 min at 4 °C. The protein concentration of lysates was measured by Bradford assay (Bio-Rad, Hercules, CA, USA). The lysates (20 µg) were resolved by 12% sodium dodecyl sulfate-polyacrylamide gel electrophoresis (SDS-PAGE) and transferred to polyvinylidene difluoride (PVDF) membranes (Millipore, Bedford, MA, USA) at 100 V for 2 h. The PVDF membranes were incubated in a blocking buffer (5% skim milk and 0.05% Tween 20 in PBS) for 2 h. The membranes were incubated with primary antibodies against MITF (Santa Cruz Biotechnology, Santa Cruz, CA, USA), tyrosinase (Santa Cruz Biotechnology, Santa Cruz, CA, USA), TRP-1 (Santa Cruz Biotechnology, Santa Cruz, CA, USA), TRP-2 (Santa Cruz Biotechnology, Santa Cruz, CA, USA), p-38 (Santa Cruz Biotechnology, Santa Cruz, CA, USA), pp-38 (Santa Cruz Biotechnology, Santa Cruz, CA, USA), p-CREB (Cell Signaling Technology, Danvers, MA, USA), CREB (Cell Signaling Technology, Danvers, MA, USA), or GAPDH (Santa Cruz Biotechnology, Santa Cruz, CA, USA) at 4 °C overnight. After being washed, the membranes were incubated with horseradish peroxidase-conjugated anti-rabbit or anti-mouse immunoglobulin secondary antibodies for 1 h 30 min. The protein band detections on the PVDF membranes were conducted by using WesternBright™ ECL reagents (Advansta Corp., Menlo Park, CA, USA) and a C300 chemiluminescence imager (Azure Biosystems, Inc., Dublin, CA, USA).

### 3.9. Statistical Analysis

All studies were repeated at least three times and results were reported as mean ± SEM. The authors used one-way ANOVA for multi-group comparisons, followed by a Tukey post hoc test; a *p*-value of less than 0.05 was considered statistically significant. Statistical differences were evaluated using Graph Pad Prism version 5.01 (GraphPad Software, San Diego, CA, USA).

## 4. Conclusions

In summary, compound **3** decreased melanin production and intracellular tyrosinase activity by modulating CREB and p38 signaling pathways in α-MSH-activated B16-F10 melanoma cells. These results showed that *M. alba* leaves could be excellent natural source for skin-whitening agents.

## Supplementary Materials

The following are available online. Isolation scheme of *M. alba* leaves and NMR spectroscopic data of isolated compounds.
