# Supplementary material for: Identification of Anti-Melanogenesis Constituents from Morus alba L. Leaves
Source: Molecules. 2018 Oct 8;23(10):2559. doi: 10.3390/molecules23102559 (PMC6222840; doi:10.3390/molecules23102559)
Supplement: Supplementary file 1 [file molecules-23-02559-s001.pdf]

# Supplementary Materials: Identification of Anti-Melanogenesis Constituents from *Morus alba* L. Leaves

**Hong Xu Li, Jung Up Park, Xiang Dong Su, Kyung Tae Kim, Jong Seong Kang, Young Ran Kim, Young Ho Kim, and Seo Young Yang**

- **Isolation scheme of *Morus alba* leaves**

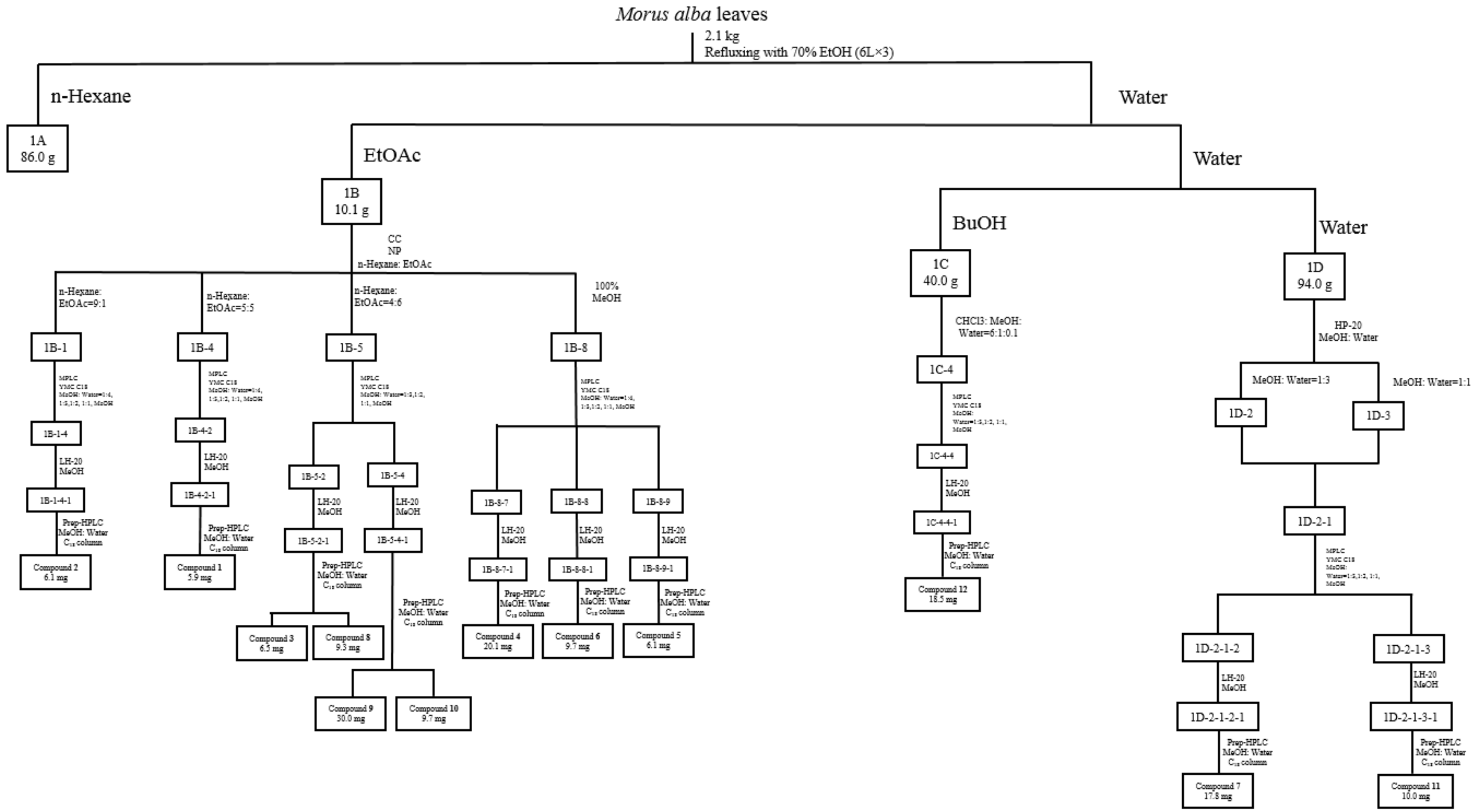

**Figure S1.** Isolation scheme of *Morus alba* leaves.

- ## NMR spectroscopic data of isolated compounds

**1.** Norartocarpetin; 2-(2,4-dihydroxyphenyl)-5,7-dihydroxy-4*H*-1-benzopyran-4-one (**1**)

$^{1}$H NMR (400 MHz, DMSO) δ 7.76(d, *J* = 8.8 Hz, 1H), 6.99 (s, 1H), 6.48 (d, *J* = 2.3 Hz, 1H), 6.43 (m, 2H), 6.16 (d, *J* = 2.1 Hz, 1H). $^{13}$C NMR (150 MHz, DMSO) δ 181.8, 163.9, 161.7, 161.7, 158.8, 158.3, 157.3, 129.7, 108.5, 108.0, 106.7, 103.5, 103.2, 98.5, 93.7.

**2.** Moracin B; 2-(3-hydroxy-5-methoxyphenyl)-6-methoxy-5-benzofuranol (**2**)

$^{1}$H NMR (300 MHz, MeOD) δ 7.14 (s, 1H), 6.95 (s, 1H), 6.92 (s, 1H), 6.85 (t, *J* = 2.27 Hz, 2H), 6.33 (t, *J* = 2.19 Hz, 1H), 3.91 (s, 3H), 3.81 (s, 3H). $^{13}$C NMR (150 MHz, MeOD) δ 162.6, 159.9, 156.2, 150.5, 148.2, 144.7, 133.9, 122.9, 106.1, 104.9, 102.5, 102.3, 102.1, 95.9, 56.7, 55.7.

**3.** Moracin J; 5-(5-hydroxy-6-methoxy-2-benzofuranyl)-1,3-benzenediol (**3**)

$^{1}$H NMR (300 MHz, MeOD) δ 7.14 (d, *J* = 2.5 Hz, 1H), 6.95 (s, 1H), 6.87 (d, *J* = 2.5 Hz, 1H), 6.75 (d, *J* = 2.2 Hz, 2H), 6.23 (t, *J* = 2.2 Hz, 1H), 3.92(s, 3H). $^{13}$C NMR (150 MHz, MeOD) δ 159.92, 156.51, 150.54, 148.19, 144.71, 133.88, 123.02, 106.15, 103.90, 103.44, 102.21, 95.96, 56.76.

**4.** Moracin M; 5-(6-hydroxy-2-benzofuranyl)-1,3-benzenediol (**4**)

$^{1}$H NMR (300 MHz, MeOD) δ 7.25 (d, *J* = 8.4 HZ, 1H), 6.81(s, 1H), 6.80 (d, *J* = 2.5 HZ, 1H), 6.67 (s, 2H), 6.66 (s, 2H), 6.63 (dd, *J* = 8.4, 2.1 HZ, 1H), 6.15 (t, *J* = 4.2, 2.1 Hz, 1H). $^{13}$C NMR (75 MHz, MeOD) δ 159.9, 157.1, 156.8, 156.0, 133.7, 123.0, 122.0, 113.2, 103.8, 103.4, 102.2, 98.4.

**5.** Moracin M 3'-*O*-β-glucopyranoside; 3-hydroxy-5-(6-hydroxy-2-benzofuranyl)phenyl-β-D-glucopyranoside (**5**)

$^{1}$H NMR (400 MHz, MeOD) δ 7.36 (d, *J* = 8.0 Hz, 1H), 7.08 (dd, *J* = 2.0, 1.0 Hz, 1H), 7.00 (d, *J* = 1.0 Hz, 1H), 6.94 (dd, *J* = 2.0, 1.0 Hz, 1H), 6.91 (dd, *J* = 2.0, 1.0 Hz, 1H), 6.74 (dd, *J* = 8.0, 2.0 Hz, 1H), 6.54 (t, *J* = 2.0 Hz, 1H), 4.96 (m, 1H). $^{13}$C NMR (150 MHz, MeOD) δ 160.6, 159.9, 157.3, 156.9, 155.6, 133.8, 122.9, 122.1, 113.3, 106.4, 105.2, 104.9, 102.7, 102.4, 98.4, 78.2, 78.0, 74.9, 71.4, 62.5.

**6.** Moracin M 6-*O*-β-D-glucopyranoside; 2-(3,5-dihydroxyphenyl)-6-benzofuranyl-β-D-glucopyranoside (**6**)

$^1$H NMR (400 MHz, MeOD) δ 7.46 (d, *J* = 8.0 Hz, 1H), 7.32 (d, *J* = 2.0 Hz, 1H), 7.04 (dd, *J* = 8.0, 2.0 Hz, 1H), 6.98 (s, 1H), 6.78 (d, *J* = 2.0 Hz, 2H), 6.26 (t, *J* = 2.0 Hz, 1H), 4.92 (m, 1H). $^{13}$C NMR (100 MHz, MeOD) δ 160.1, 157.4, 157.4, 156.7, 133.6, 125.5, 122.0, 121.9, 114.9, 114.9, 104.2, 104.1, 104.1, 103.9, 103.8, 103.2, 103.0, 102.1, 102.0, 100.7, 100.6, 78.2, 77.9, 74.9, 71.4, 62.5.

**7.** Mulberroside F; 3-[6-(β-D-glucopyranosyloxy)-2-benzofuranyl]-5-hydroxyphenyl-β-D-glucopyranoside (**7**)

$^1$H NMR (400 MHz, MeOD) δ 7.52 (d, *J* = 8.0 Hz, 1H), 7.32 (s, 1H), 7.27 (s, 1H), 7.01 (s, 1H), 6.98 (d, *J* = 8.0 Hz, 1H), 6.93 (s, 1H), 6.47 (m, 1H), 4.92 (d, *J* = 6.6 Hz, 1H), 4.87 (d, *J* = 7.2 Hz, 1H). $^{13}$C NMR (100 MHz, MeOD) δ 159.3, 158.9, 155.9, 154.9, 154.9, 131.5, 123.2, 121.2, 113.8, 105.0, 104.1, 103.7, 102.1, 101.1, 100.8, 99.2, 77.2, 76.7, 73.3, 69.8, 69.8, 60.8, 60.7.

**8.** Steppogenin; (2*S*)- 2-(2,4-dihydroxyphenyl)-2,3-dihydro-5,7-dihydroxy-4*H*-1-benzopyran-4-one (**8**)

$^1$H NMR (400 MHz, MeOD) δ 7.24 (d, *J* = 8.0 Hz, 1H), 6.33 (m, 2H), 5.91 (d, *J* = 2.0 Hz, 1H), 5.88 (d, *J* = 2.0 Hz, 1H), 5.60 (dd, *J* = 13.0, 2.7 Hz, 1H), 3.07 (dd, *J* = 17.0, 13.0 Hz, 1H), 2.69 (dd, *J* = 17.0, 2.7 Hz, 1H). $^{13}$C NMR (100 MHz, MeOD) δ 198.6, 168.4, 165.6, 165.5, 159.8, 156.9, 128.9, 117.9, 107.7, 103.3, 96.9, 96.1, 75.9, 43.1.

**9.** Astragalin; 3-(β-D-glucopyranosyloxy)-5,7-dihydroxy-2-(4-hydroxyphenyl)-4*H*-1-benzopyran-4-one (**9**)

$^1$H NMR (400 MHz, MeOD) δ 8.05 (d, *J* = 7.0 Hz, 2H), 6.89 (d, *J* = 7.0 Hz, 2H), 6.44(s, 1H), 6.22(s, 1H), 5.47(d, *J* = 7.0 Hz, 1H). $^{13}$C NMR (100 MHz, MeOD) δ 179.5, 166.0, 163.0, 161.6, 159.1, 158.5, 135.5, 132.3, 122.7, 116.1, 105.7, 104.1, 104.1, 99.8, 94.7, 78.3, 77.9, 75.7, 71.2, 62.5.

**10.** Isoquercitrin; 2-(3,4-dihydroxyphenyl)-3-(β-D-glucopyranosyloxy)-5,7-dihydroxy-4*H*-1-benzopyran-4-one (**10**)

$^1$H NMR (400 MHz, MeOD) δ 7.71 (d, *J* = 2.0 Hz, 1H), 7.59 (dd, *J* = 8.5, 2.0 Hz, 1H), 6.87 (d, *J* = 8.5 Hz, 1H), 6.39 (d, *J* = 2.0 Hz, 1H), 6.20 (d, *J* = 2.0 Hz, 1H), 5.26 (d, *J* = 7.5 Hz, 1H). $^{13}$C NMR (100 MHz, MeOD) δ 179.4, 166.0, 162.9, 158.9, 158.3, 149.7, 145.8, 135.4, 123.1, 123.0, 122.9, 117.4, 117.3, 115.8, 115.8, 105.5, 104.1, 104.0, 99.7, 99.7, 94.5, 78.2, 77.9, 75.5, 70.9, 62.3.

**11.** Rutin; 3-[[6-*O*-(6-deoxy-α-L-mannopyranosyl)-β-D-glucopyranosyl]oxy]-2-(3,4-dihydroxyphenyl)-5,7-dihydroxy-4*H*-1-benzopyran-4-one (**11**)

$^{1}H$ NMR (400 MHz, MeOD) δ 7.53 (m, 2H), 6.84 (d, *J* = 8.2 Hz, 1H), 6.39 (d, *J* = 1.7 Hz, 1H), 6.20 (d, *J* = 1.7 Hz, 1H), 5.34 (d, *J* = 6.6 Hz, 1H), 4.36 (d, *J* = 3.3 Hz, 1H). $^{13}C$ NMR (100 MHz, MeOD) δ 177.57, 164.27, 161.39, 156.79, 156.58, 148.58, 144.92, 133.43, 121.72, 121.29, 116.38, 115.36, 104.06, 101.27, 100.86, 98.78, 93.69, 76.50, 75.97, 74.13, 71.90, 70.61, 70.43, 70.06, 68.31, 67.04, 17.75.

**12.** Morin 3-*O*-β-D-glucopyranoside; 2-(2,4-dihydroxyphenyl)-3-(β-D-glucopyranosyloxy)-5,7-dihydroxy-4*H*-1-benzopyran-4-one (**12**)

$^{1}H$ NMR (400 MHz, DMSO) δ 7.58 (m, 2H), 6.84 (d, *J* = 8.9 Hz, 1H), 6.39 (s, 1H), 6.19 (s, 1H), 5.46 (d, *J* = 5.7 Hz, 1H). $^{13}C$ NMR (100 MHz, DMSO) δ 177.5, 164.9, 161.4, 156.5, 156.2, 148.7, 145.0, 133.4, 121.7, 121.2, 116.2, 115.3, 103.8, 101.0, 98.9, 93.6, 77.6, 76.5, 74.1, 70.0, 61.0.
